# Impact of new X-ray technology on patient dose in pacemaker and implantable cardioverter defibrillator (ICD) implantations

**DOI:** 10.1007/s10840-016-0200-z

**Published:** 2016-10-26

**Authors:** Joris D. van Dijk, Jan Paul Ottervanger, Peter Paul H. M. Delnoy, Martine C. M. Lagerweij, Siert Knollema, Cornelis H. Slump, Pieter L. Jager

**Affiliations:** 1 Department of Nuclear Medicine, Isala Hospital, PO Box 10400, 8000 GK Zwolle, The Netherlands; 2 MIRA: Institute for Biomedical Technology and Technical Medicine, University of Twente, Enschede, The Netherlands; 3 Department of Cardiology, Isala Hospital, Zwolle, The Netherlands; 4 Department of Medical Physics, Isala Hospital, Zwolle, The Netherlands

**Keywords:** Dose reduction, Imaging, Radiation dose, Pacemaker, Implantable cardioverter defibrillator (ICD)

## Abstract

**Purpose:**

New X-ray technology providing new image processing techniques may reduce radiation exposure. The aim of this study was to quantify this radiation exposure reduction for patients during pacemaker and implantable cardioverter defibrillator (ICD) implantation.

**Methods:**

In this retrospective study, 1185 consecutive patients who had undergone *de novo* pacemaker or ICD implantation during a 2-year period were included. All implantations in the first year were performed using the reference technology (Allura Xper), whereas in the second year, the new X-ray technology (AlluraClarity) was used. Radiation exposure, expressed as the dose area product (DAP), was compared between the two time periods to determine the radiation exposure reduction for pacemaker and ICD implantations without cardiac resynchronization therapy (CRT) and with CRT. Procedure duration and contrast volume were used as measures to compare complexity and image quality.

**Results:**

The study population consisted of 591 patients who had undergone an implantation using the reference technology, and 594 patients with the new X-ray technology. The two groups did not differ in age, gender, or body mass index. The DAP decreased with 69 % from 16.4 ± 18.5 to 5.2 ± 6.6 Gy cm^2^ for the non-CRT implantations (*p* < 0.001). The DAP decreased with 75 % from 72.1 ± 60.0 to 17.8 ± 17.4 Gy cm^2^ for the CRT implantations (*p* < 0.001). Nevertheless, procedure duration and contrast volume did not differ when using the new technology (*p* = 0.09 and *p* = 0.20, respectively).

**Conclusions:**

Introduction of new X-ray technology resulted in a radiation exposure reduction of more than 69 % for patients during pacemaker and ICD implantation while image quality was unaffected.

## Introduction

The use of pacemakers and implantable cardioverter defibrillator (ICD) has increased sharply over the past decade [1]. This increase has led to concerns about the long-term health consequences of the radiation exposure during the implantation of these devices [2]. Patients and in particular staff can be exposed to high cumulative doses due to the increasing complexity and high number of pacemaker and ICD implantations. Minimizing the radiation exposure while maintaining an acceptable image quality is therefore essential, especially for staff performing many procedures every year [3, 4].

New imaging technologies have the potential to decrease radiation exposure while maintaining image quality. This is mainly due to ongoing developments in both computational power and software algorithms [5]. A new X-ray imaging technology (AlluraClarity), commercially introduced during in mid-2012, is equipped with the latest image processing techniques, showed in pre-clinical setting radiation exposure decreases of between 50 and 85 % [5]. These results were confirmed for coronary angiography and electrophysiology procedures in clinical practice [6–8]. However, these results have not been confirmed for pacemaker and ICD implantations where different image processing settings and projection angles are used compared to coronary angiography or electrophysiological procedures. The aims of this study were to quantify the reduction in radiation exposure for patients during pacemaker or ICD implantation using the new AlluraClarity X-ray technology, and to assess whether image quality was comparable to that achieved previously.

## Methods

### Study population

This was a retrospective cohort study. All patients who underwent *de novo* implantation of a pacemaker or ICD in our institution between August 16, 2012 and August 16, 2014 were included. The standard acquisition chain and image processing (hereafter, referred to as the reference technology, Allura Xper FD10, Philips Healthcare) were used during all implantations performed in the first year (<August 16, 2013). A new X-ray technology (hereafter, referred to as the new technology, AlluraClarity FD10, Philips Healthcare) was used in all implantations performed in the second year. The new X-ray equipment has more computational power than conventional X-ray equipment and therefore features real-time automatic motion compensation to align moving structures before averaging. This correction allows averaging over more consecutive images resulting in an increased temporal noise reduction. Moreover, the new hardware also allows to average the intensity of more neighborhood pixels in a single frame than the conventional systems, improving the spatial noise reduction. Finally, new imaging algorithms also improve brightness control and edge and contrast enhancement.

### Procedure

The indication for a pacemaker or ICD implantation was determined according to the European guidelines at the time of implantation [9, 10]. Pacemaker and ICD devices from any of the five major manufacturers (Medtronic Inc, St Jude Medical, Boston-Scientific, Biotronik, and Sorin Group) were implanted. All the implants were inserted through a pectoral incision, and the leads were inserted through the subclavian vein. The majority of coronary sinus leads was bipolar and was positioned in the lateral, posterolateral, or posterior region wherever possible, whereas the anterior and anterolateral positions were considered suboptimal and avoided if possible. Fluoroscopic guidance was used to ensure accurate tip placement. The standard image processing settings for pacemaker and ICD implantations were used for both reference and new X-ray technologies, as advised by the vendor, and are shown in Table 1. This also included the lower radiation exposure settings when using the new technology. All procedures were started using the default “low” fluoroscopy dose setting and were increased by the cardiologist from a “low” to “medium” and from a “medium” to “high” exposure rate when clinically indicated.

**Table 1:** Standard fluoroscopic and cinematographic imaging settings—including an example of the tube settings for a typical patient corresponding to a 20-cm equivalent water thickness (with a constant source-to-image receptor distance of 87 cm, field size of 25 mm without magnification)—for both the reference technology (Alura Xper) and new X-ray technology (AlluraClarity)

Setting	Reference technology			New technology		
	Low	Medium	High	Low	Medium	High
Cu filtering (mm)	0.9	0.9	0.4	0.4	0.4	0.1
Al-filtering (mm)	1.0	1.0	1.0	1.0	1.0	1.0
Detector dose rate (nGy/s)	310	660	720	100	200	230
Entrance dose limitation (μGy/s)	140	349	697	75	145	365
Frame rates						
Fluoroscopy (frames/s)	15	15	15	7.5	7.5	7.5
Cineangiography (frames/s)	3.75	7.5	15	3.75	7.5	15
Fluoroscopy (20-cm water equivalent)						
Tube voltage (kV)	96	93	83	84	85	76
Tube current (mA)	2.6	6.9	7.2	0.9	1.8	4.0

The dose area product (DAP) was measured by the ionization chambers inside the X-ray systems, and the cumulative DAP was derived for each procedure. We distinguished between two types of implantations: the standard pacemaker or ICD devices and the implantations with cardiac resynchronization therapy (CRT), all with biventricular lead placements. The latter procedure is generally associated with a higher radiation exposure and use of contrast fluids, possibly influencing the radiation exposure reduction when introducing the new technology. The mean DAP was compared between the two X-ray technologies for both groups to determine differences in radiation exposure. In addition, the procedure time and contrast volume were also compared between the two technologies for both type of implantations as measures of image quality and procedural complexity. Procedure time was defined as the total occupation time of the operating room, including room preparation and the time-out procedure. Influence of operator experience on the radiation exposure reduction was assessed. An experienced operator was defined as an operator performing more than 80 procedures in the 2-year period. In addition, we assessed a possible learning curve for using the new technology by comparing the mean DAP in the first 3 months after installation of the new technology with the mean DAP in the last 3 months.

### Statistics

All patient-specific parameters and characteristics for both the groups were presented as percentages or mean ± standard deviation (sd), and compared using the chi-square or unpaired *t* tests as appropriate, using Stata software (StataSE 12.0). The radiation exposure, expressed as the DAP, and procedure time were compared between the two technologies using a *t* test, for both the non-CRT devices and CRT devices. The same test was used to test for difference in contrast volume for the CRT devices between both the technologies. Percentage of procedures performed by experienced operators was compared between the two technologies using a *t* test. Influence of operator experience on the reduction in radiation exposure was tested using a two-way ANOVA for the non-CRT and CRT implantations. The mean radiation exposure in the first 3 months was compared with the last 3 months for the new technology by using a *t* test. The level of statistical significance was set to 0.05 for all the statistical analyses.

## Results

A total of 1185 patients were included in this study. The baseline characteristics are summarized in Table 2. The study population consisted of 591 patients who underwent implantation using the reference technology, and 594 patients on whom the new X-ray technology was used. Both the groups were comparable regarding age, gender, body weight, body mass index, and percentage of ICD implantations.

**Table 2:** Demographics of all the patients included in the study who underwent pacemaker or ICD implantation using either the reference or the new X-ray technology

Characteristic	Reference technology (*n* = 591)	New technology (*n* = 594)	*p* value
Age (years)	70.2 ± 12.1	69.9 ± 11.9	0.61
Male gender (%)	66.2	65.4	0.72
Body mass (kg)	84.8 ± 17.0	84.3 ± 15.1	0.69
Height (cm)	174 ± 9.0	174 ± 9.5	0.77
BMI (kg/m^2^)	27.8 ± 4.9	27.8 ± 4.6	0.92
ICD (%)	50.4	55.2	0.09

Data are presented as mean ± standard deviation, or as percentages

**Fig. 1:**
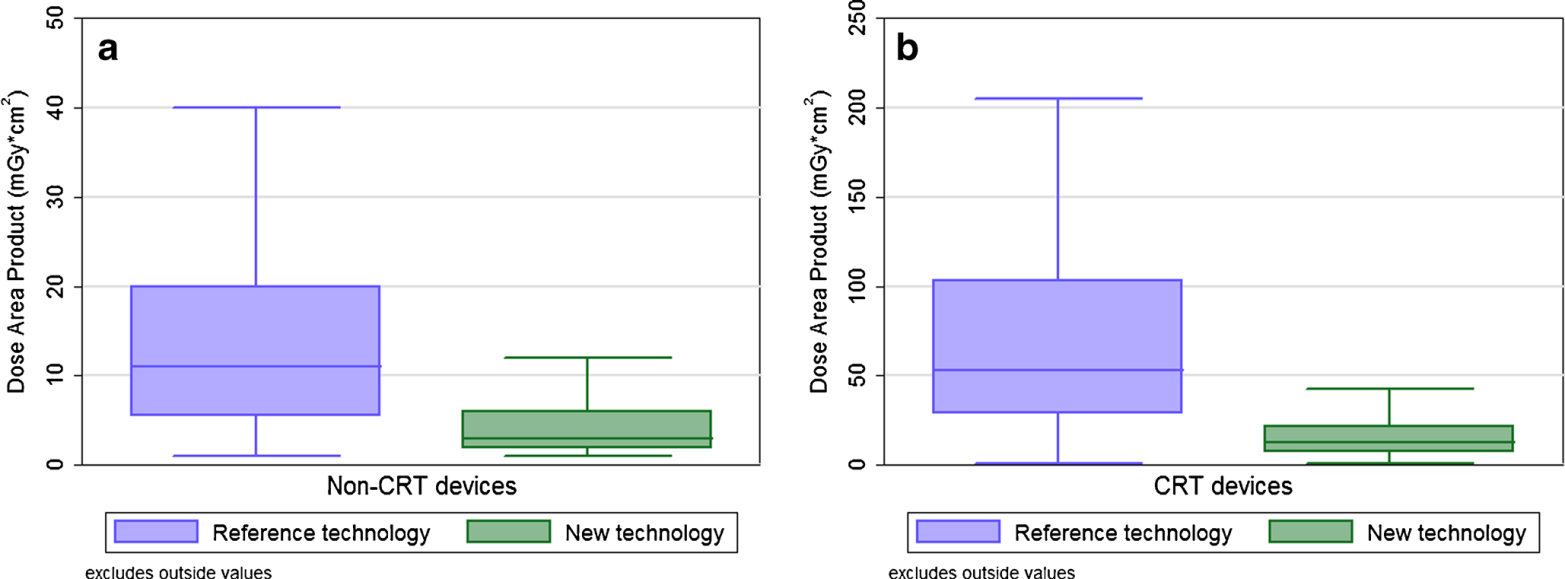
Boxplot showing the radiation exposure—expressed as the dose area product—during pacemaker or ICD implantation for the non-cardiac resynchronization therapy devices (CRT) (**a**) and CRT devices (**b**) when using either the reference (*n* = 591) or new X-ray technology (*n* = 594). Radiation exposure decreased with 69 % for the non-CRT and 75 % for the CRT implantations (*p* < 0.001). Outlier values, defined as 1.5 times the interquartile range, are not shown

The percentage of CRT implantations differed between the two patient groups. The percentage of patients who received a CRT device was 31 % (183) using the reference technology versus 39 % (230) using the new technology (*p* = 0.005). For the non-CRT implantations, the mean cumulative radiation exposure reduction was 69 % when using the new X-ray technology, as shown in Fig. 1. The mean DAP decreased during these procedures from 16.4 ± 18.5 to 5.2 ± 6.6 Gy cm^2^ (*p* < 0.001). The radiation exposure reduction when implanting CRT devices was 75 %. The mean DAP decreased from 72.1 ± 60.0 to 17.8 ± 17.4 Gy cm^2^ for these implantations (*p* < 0.001).

**Fig. 2:**
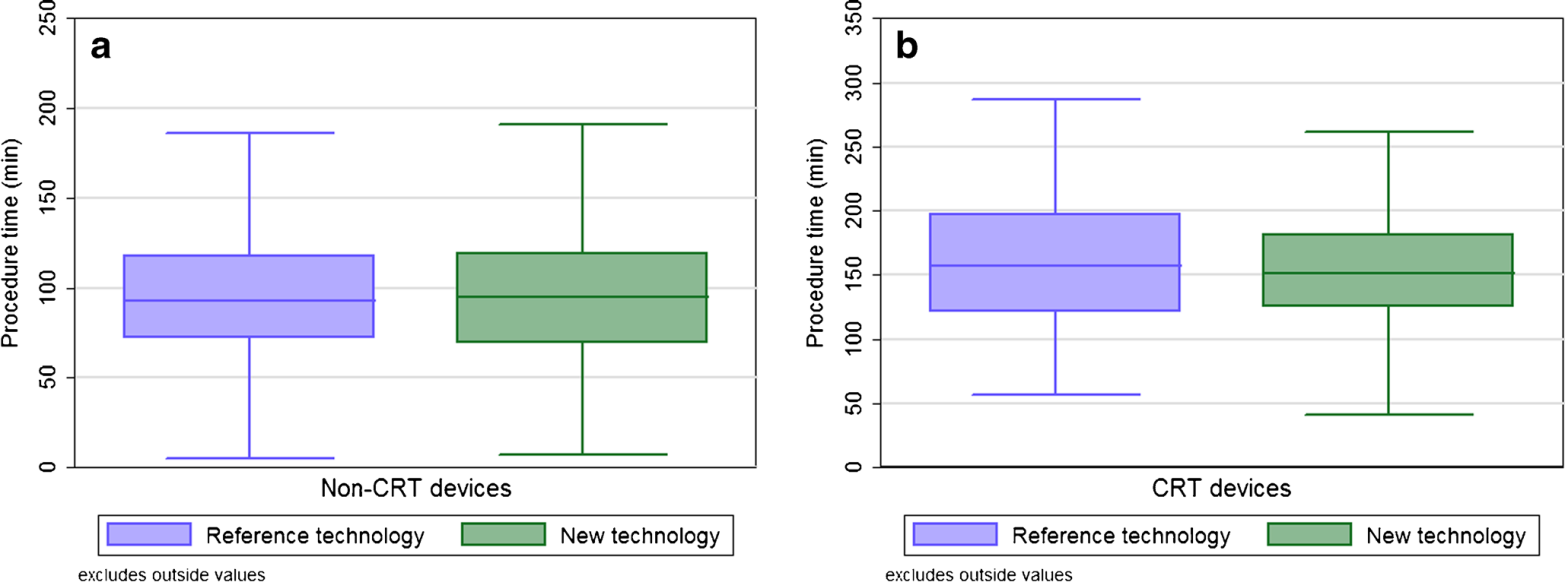
Boxplots showing the procedure time for the non-cardiac resynchronization therapy (CRT) devices (*p* = 0.68) (**a**) and CRT devices (*p* = 0.09) (**b**) when using the reference or the new X-ray technology. Outlier values, defined as 1.5 times the interquartile range, are not shown

**Fig. 3:**
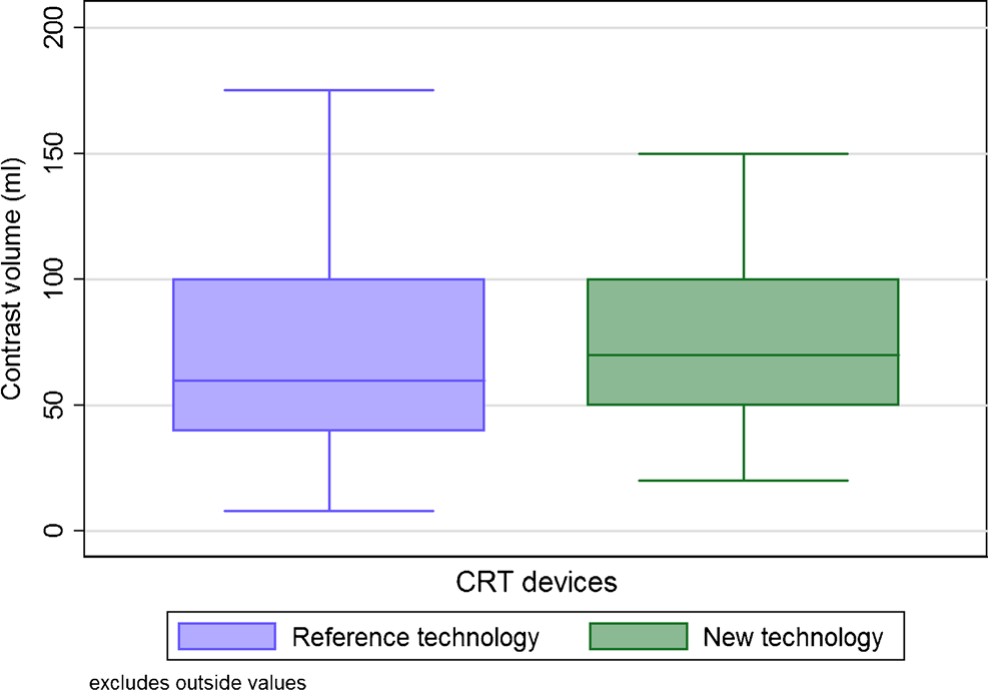
Boxplot showing the volume of injected contrast used during implantation of the CRT devices when using the reference or the new X-ray technology (*p* = 0.20). Outlier values, defined as 1.5 times the interquartile range, are not shown

Despite these reductions, the procedure time did not differ between using the reference technology (96.6 ± 47 and 162.6 ± 52.7 min for the CRT implantations) and the new technology (98.0 ± 47.3 and 154.2 ± 47.2 min for the CRT implantations), for both the non-CRT and CRT implantations (*p* = 0.68 and *p* = 0.09, respectively), as shown in Fig. 2. The mean volume of contrast injected during the CRT procedures did not differ between using the reference technology (75 ± 56 ml) and the new X-ray technology (83 ± 52 ml, *p* = 0.20), as shown in Fig. 3.

The percentage of procedures performed by experienced operators did not differ between the two groups. The non-CRT implantations were performed by experienced operators using the reference and new technologies in 48 and 46 % of the cases (*p* = 0.71). The percentage of the CRT procedures performed by experienced physician was higher: 60 and 54 % using the reference and new technologies (*p* = 0.24). Operator experience did not influence the reduction in radiation exposure for the non-CRT implantations (*p* = 0.87) or for the CRT implantations (*p* = 0.08).

A learning curve using the new technology was absent comparing the mean radiation exposure in the first 3 months and the last 3 months for the non-CRT implantations (*p* = 0.15). However, the radiation exposure decreased from 18.1 ± 16.8 to 12.7 ± 10.5 Gy cm^2^ for the CRT implantations after 9 months (*p* = 0.04).

## Discussion

In this study, we have shown that the use of the new X-ray technology, with its new processing algorithms and hardware, reduces the average used radiation exposure for patients by over 69 % during both pacemaker and ICD implantations. Despite this radical reduction, the procedure time and used contrast volume, both indirect measures of image quality, did not change.

The observed reduction in radiation exposure is in the same range as the reductions reported by previous studies. One study assessing the exposure reduction in complex electrophysiologic procedures reported a dose reduction of 40 % without compromising image quality [8]. Another study reported an exposure reduction of 75 % in coronary angiography procedures while maintaining image quality [7]. In addition, a large retrospective study reported an exposure reduction of 66 % when using fluoroscopy and cineangiography in coronary angiography and percutaneous interventions [6]. Moreover, Söderman et al. reported an exposure reduction of 60 % in neuroradiology and interventional neuroradiology when using both fluoroscopy and digital subtraction angiography [11]. However, the same group reported a reduction of 75 % when only using digital subtraction angiography, indicating a smaller reduction when using only fluoroscopy [12]. This is in agreement with the reported exposure reduction of 83 % in iliac artery digital subtraction angiography procedures [13].

It seems that in these studies, the reported radiation exposure reduction is higher using digital subtraction angiography in comparison to that achieved with fluoroscopy and/or cinematography. The variation in the radiation exposure reduction using fluoroscopy and/or cineangiography is probably mainly due to the variation in image processing settings. For each procedure, the optimal combination of the four main image processing technologies incorporated in the new technology is used: real-time pixel shift, motion compensation, noise reduction, and image enhancement [5]. This should provide the optimal image quality for each procedure, and these different settings will most probably influence the reduction in radiation exposure, explaining the differences between studies.

The mean DAP measured in this study using the reference technology is comparable to typical values in literature. The DAP for the non-CRT devices in this study, 16.4 Gy cm^2^, was even lower than that for the typical value as reported by Heidbuchel et al.: 4 mSv, corresponding to a DAP of 20 Gy cm^2^ [14]. The same tendency was observed for CRT devices with a mean DAP of 72.1 Gy cm^2^ in this study in comparison to the typical values of 110 Gy cm^2^ (22 mSv) as mentioned in the practical guide to reduce radiation dose [14].

Beside the AlluraClarity system (Philips Healthcare), which is described in this study, the other major vendors also introduced new angiographic systems last years, focused on dose reduction while maintaining or increasing image quality (GE Healthcare, IGS 730 and IGS 740; Siemens, Artis Q.zen; and Toshiba, Infinix Elite). However, only one conference proceeding has been published so far describing a dose reduction in clinical practice. They reported a radiation exposure reduction of 55 % using Artis Q.zen technology [15]. Moreover, Christopoulos et al. performed a bench test with an anthropomorphic phantom comparing the radiation dose of the new AlluraClarity system with three other fluoroscopy systems [16]. They compared the DAP and showed that the new technique resulted DAP reductions of 74, 69, and 48 % in comparison to the Innova IGS (GE Healthcare), Artis One (Siemens), and Integris Allura FD20 (Philips), respectively. Yet their study did not include the new techniques from the other two main vendors. It is therefore still unknown how the new technologies of the other vendors compare in cardiac procedures to the technology evaluated in this study.

Introduction of the new technology improves the radiation safety for both patients and staff in the operating rooms. Despite the relatively low radiation exposure associated with ICD and pacemaker implantations using the new technology, procedures still should be performed in accordance with the as low as reasonably achievable (ALARA) principle and should be in compliance with the latest procedure indications as stated in the guidelines. Further refinement of standard procedure settings, such as decreasing fluoroscopy frame rates or cine acquisitions, introduction of body weight depending exposure protocols, and increasing the awareness of the patients’ dose associated with the various projection angles, could help in further minimizing the required radiation exposure [14]. Moreover, introduction non-fluoroscopic mapping systems using electromagnetic guidance could result in additional dose reductions [14].

Several assumptions underpinned this study. First, we used a retrospective study design. However, we expect this influence to be minimal due to the consecutive nature of the inclusion, the large number of patients as well as the fact that no additional radiation exposure reduction measures were introduced during the inclusion period. Second, only indirect measures of image quality—procedure time and contrast volume—were assessed. Yet both indirect measures were identical between both the technologies for the non-CRT and CRT implantations. In addition, operators were able to manually increase the fluoroscopy dose setting to ensure that a sufficient image quality was obtained for all the patients. Third, the observed radiation exposure reduction is not only due to the new image processing technologies. The new technology also allows storage of the fluoroscopy and cineangiography acquisitions. The ability to replay these acquisitions during the procedures obviates the need to subject the patient to additional radiation. Although we were not able to quantify the possible decrease in fluoroscopy time or cineangiography acquisitions as these are not routinely recorded, we can reasonably assume this storage function also contributed to the observed radiation exposure reduction. Fourth, regional hospitals refer patients with complex anatomy and pathology to our tertiary hospital. This higher patient complexity results in the use of higher volumes of contrast fluids and increased procedure times. However, used contrast volume and procedure complexity were comparable between the two periods, and the higher procedure complexity was therefore not expected to influence the outcomes. Final, the effect of the new X-ray technology on the staff radiation dose was not assessed as no additional dosage or exposure measurements were registered for individual procedures directly at the staff. However, it can reasonably be assumed that the staff radiation dose, caused by scattered radiation, is proportional to the patient dose, as shown previously [8]. Hence, we can assume that the lower patient radiation exposure will also result in a comparable dose reduction for the staff.

## Conclusion

Introduction of new X-ray technology resulted in radiation exposure reductions of 69 % in the non-cardiac resynchronization therapeutic cardiac pacemaker and ICD implantations and 75 % in the cardiac resynchronization therapeutic device implantations while image quality was unaffected.

## References

[1] Boriani G, Berti E, Belotti LMB, Biffi M, Carboni A, Bandini A (2014). Cardiac resynchronization therapy. J. Cardiovasc. Med.

[2] International Commission on Radiological Protection (ICRP).(2000). Avoidance of radiation injuries from medical interventional procedures. ICRP Publ. 85. Ann. ICRP 30. 2000 10.1016/S0146-6453(01)00004-5 11459599

[3] Prasad KN (2004). Radiation protection in humans: extending the concept of as low as reasonably achievable (ALARA) from dose to biological damage. Br J Radiol.

[4] Fahey F, Stabin M (2014). Dose optimization in nuclear medicine. Semin Nucl Med.

[5] Philips Healthcare. ClarityIQ technology—white paper. Available at: www.forms.healthcare.philips.com. (Accessed on: May 1, 2015)

[6] Nakamura, S., Kobayashi, T., Funatsu, A., Okada, T., Mauti, M., Waizumi, Y., et al.(2015). Patient radiation dose reduction using an X-ray imaging noise reduction technology for cardiac angiography and intervention. Heart and vessels, 4 apr. Epub ahead of print. 10.1007/s00380-015-0667-z 25840815

[7] Eloot L, Thierens H, Taeymans Y, Drieghe B, De Pooter J, Van Peteghem S (2015). Novel X-ray imaging technology enables significant patient dose reduction in interventional cardiology while maintaining diagnostic image quality. Catheter Cardiovasc Interv.

[8] Dekker, L.R.C., van der Voort, P.H., Simmers, T.A., Verbeek, X.A., Bullens, R.W.M., Veer, M.V. et al.(2013). New image processing and noise reduction technology allows reduction of radiation exposure in complex electrophysiologic interventions while maintaining optimal image quality: a randomized clinical trial. Heart rhythm : the official journal of the Heart Rhythm Society, 10 (11), 1678–82 10.1016/j.hrthm.2013.08.018 23973946

[9] Vardas PE, Auricchio A, Blanc J-J, Daubert J-C, Drexler H, Ector H (2007). Guidelines for cardiac pacing and cardiac resynchronization therapy: the Task Force for cardiac pacing and cardiac resynchronization therapy of the European Society of Cardiology. Developed in collaboration with the European Heart Rhythm Association. Eur Heart J.

[10] Brignole M, Auricchio A, Baron-Esquivias G, Bordachar P, Boriani G, Breithardt O-A (2013). 2013 ESC guidelines on cardiac pacing and cardiac resynchronization therapy: the Task Force on cardiac pacing and resynchronization therapy of the European Society of Cardiology (ESC). Developed in collaboration with the European Heart Rhythm Association. Eur Heart J.

[11] Söderman M, Mauti M, Boon S, Omar A, Marteinsdóttir M, Andersson T (2013). Radiation dose in neuroangiography using image noise reduction technology: a population study based on 614 patients. Neuroradiology.

[12] Söderman M, Holmin S, Andersson T, Palmgren C, Babic D, Hoornaert B (2013). Image noise reduction algorithm for digital subtraction angiography: clinical results. Radiology.

[13] van Strijen MJ, Grünhagen T, Mauti M, Zähringer M, Gaines PA, Robinson GJ (2015). Evaluation of a noise reduction imaging technology in iliac digital subtraction angiography: noninferior clinical image quality with lower patient and scatter dose. J Vasc Interv Radiol.

[14] Heidbuchel H, Wittkampf FHM, Vano E, Ernst S, Schilling R, Picano E (2014). Practical ways to reduce radiation dose for patients and staff during device implantations and electrophysiological procedures. Europace.

[15] Anderson B, Lee S, Vuong D, Justo R, Ward C (2015). Effects of Siemens Artis Q.zen on radiation dose on patent ductus arteriosus closure: a single centre experience. Heart Lung Circ.

[16] Christopoulos G, Christakopoulos GE, Rangan BV, Layne R, Grabarkewitz R, Haagen D (2015). Comparison of radiation dose between different fluoroscopy systems in the modern catheterization laboratory: results from bench testing using an anthropomorphic phantom. Catheter Cardiovasc Interv.

